# Urbanization, mainly rurality, but not altitude is associated with dyslipidemia profiles

**DOI:** 10.1016/j.jacl.2017.06.016

**Published:** 2017

**Authors:** Maria Lazo-Porras, Antonio Bernabe-Ortiz, Renato Quispe, German Málaga, Liam Smeeth, Robert H. Gilman, William Checkley, J. Jaime Miranda, CRONICAS Cohort Study Group

**Affiliations:** a CRONICAS Center of Excellence in Chronic Diseases, Universidad Peruana Cayetano Heredia, Lima, Peru; b CONEVID Unidad de Conocimiento y Evidencia, Universidad Peruana Cayetano Heredia, Lima, Peru; c Faculty of Epidemiology and Population Health, London School of Hygiene and Tropical Medicine, London, United Kingdom; d Department of International Health, Bloomberg School of Public Health, Johns Hopkins University, Baltimore, MD, USA; e Área de Investigación y Desarrollo, Asociación Benéfica PRISMA, Lima, Peru; f Division of Pulmonary and Critical Care, School of Medicine, Johns Hopkins University, Baltimore, MD, USA; g Department of Medicine, School of Medicine, Universidad Peruana Cayetano Heredia, Lima, Peru

**Keywords:** Urbanization, Rurality, Altitude, Dyslipidemia, Environment

## Abstract

**Background:**

Geographical and environmental features such as urbanization and altitude may influence individual's lipid profiles because of the diversity of human-environment interactions including lifestyles.

**Objective:**

To characterize the association between altitude and urbanization and lipid profile among Peruvian adults aged ≥35 years.

**Methods:**

Cross-sectional analysis of the CRONICAS Cohort Study. The outcomes of interest were 6 dyslipidemia traits: hypertriglyceridemia, high low-density lipoprotein cholesterol, low high-density lipoprotein cholesterol (HDL-c), nonisolated low HDL-c, isolated low HDL-c, and high non-HDL-c. The exposures of interest were urbanization level (highly urban, urban, semi-urban, and rural) and altitude (high altitude vs sea level). Prevalence ratios (PRs) and 95% confidence intervals (95% CIs) were calculated using Poisson regression models with robust variance adjusting for potential confounders.

**Results:**

Data from 3037 individuals, 48.5% males, mean age of 55.6 (standard deviation ±12.7) years, were analyzed. The most common dyslipidemia pattern was high non-HDL-c with a prevalence of 88.0% (95% CI: 84.9%–90.7%) in the rural area and 96.0% (95% CI: 94.5%–97.1%) in the semi-urban area. Relative to the highly urban area, living in rural areas was associated with a lower prevalence of hypertriglyceridemia (PR = 0.75; 95% CI: 0.56–0.99) and high non-HDL-c (PR = 0.96; 95% CI: 0.93–0.99), whereas living in semi-urban areas was associated with higher prevalence high low-density lipoprotein cholesterol (PR = 1.37; 95% CI: 1.11–1.67). Compared with sea level areas, high-altitude areas had lower prevalence of high non-HDL-c (PR = 0.97; 95% CI: 0.95–0.99).

**Conclusion:**

Urbanization but not altitude was associated to several dyslipidemia traits, with the exception of high non-HDL-c in high altitude settings.

## Highlights

• Levels of urbanization but not altitude were associated with dyslipidemia traits.
• People in rural in comparison to urban areas had lower prevalence of dyslipidemia.
• Population from semi-urban areas showed high prevalence of some dyslipidemia traits.

## Introduction

Although cardiovascular diseases (CVDs) continue being the leading cause of death worldwide, the prevalence of dyslipidemia–the key underlying process contributing to most CVD–continues increasing worldwide.^1^ Indeed, high prevalence of dyslipidemia has been reported in low- and middle-income countries, especially in Latin America, such as 57% of individuals with low levels of high-density lipoprotein cholesterol (HDL-c) in Lima, 32% of hypertriglyceridemia (high triglyceride [TG]) in Mexico city, and 24% of high low-density lipoprotein-cholesterol (LDL-c) levels in Buenos Aires.^2^

Levels of serum lipids are influenced by several environmental factors. For instance, fatty acid and carbohydrate content and composition in diet, adiposity, physical activity, and alcohol intake have been shown to be important determinants of lipoprotein secretion and metabolism.^3^ However, the role of environmental factors directly related to the place of residence, such as urbanization or altitude, has not been fully understood.

The potential association between high altitude and dyslipidemia remains controversial. Some studies in Peru have found a positive association between high altitude with hypertriglyceridemia and low HDL-c.^4^, ^5^ Also, a study conducted in Lhasa, Tibet, located at 3660 meters above sea level (m.a.s.l.), found a high prevalence of hypertriglyceridemia and low concentrations of HDL-c.^6^ On the other hand, one study in Peru reported a low prevalence of hypercholesterolemia, hypertriglyceridemia, and low HDL-c in high altitude (>3000 m.a.s.l.) compared with sea level population.^7^ Another study in Arab populations found higher levels of HDL-c in people who live at 2000 m.a.s.l. relative to those who live at sea level.^8^ These studies denote that the controversial results in the association between high altitude and dyslipidemia patterns and does not account for the rural/urban effect that is also be present even at different altitudes.

Studying the isolated effect of high altitude and urbanization on lipid levels is challenging, as both are strongly associated with different lifestyle behaviors compared with sea level and rural counterparts, respectively. For example, lifestyle in rural areas includes a dietary intake characterized by high levels of carbohydrates^9^, ^10^ and greater levels of physical activity compared with urban areas.^11^ Understanding the diversity of human-environment interactions with regard to dyslipidemias is important, especially if in 2014, 88% of Latin America and Caribbean population lived in urban areas,^12^ and it is calculated that 35 million people live above 2500 m.a.s.l in South America between the cities of Bolivia, Colombia, Ecuador, and Peru.^13^ Also, between 10 and 17 million people live at over 2500 m.a.s.l. in the Andes.^14^

Previous studies in dyslipidemias were predominantly conducted in rural areas in high-altitude or urban sea level cities. The CRONICAS Cohort Study was designed to evaluate Peruvian adults from 4 settings differing on the levels of altitude and urbanization, allowing for combinations of rural-urban and sea level-high altitude settings. As such, it offers a unique opportunity to test our hypothesis and characterize the association between altitude and urbanization and lipid profiles.

## Methods

### Study design, settings, and participants

**Figure 1:**
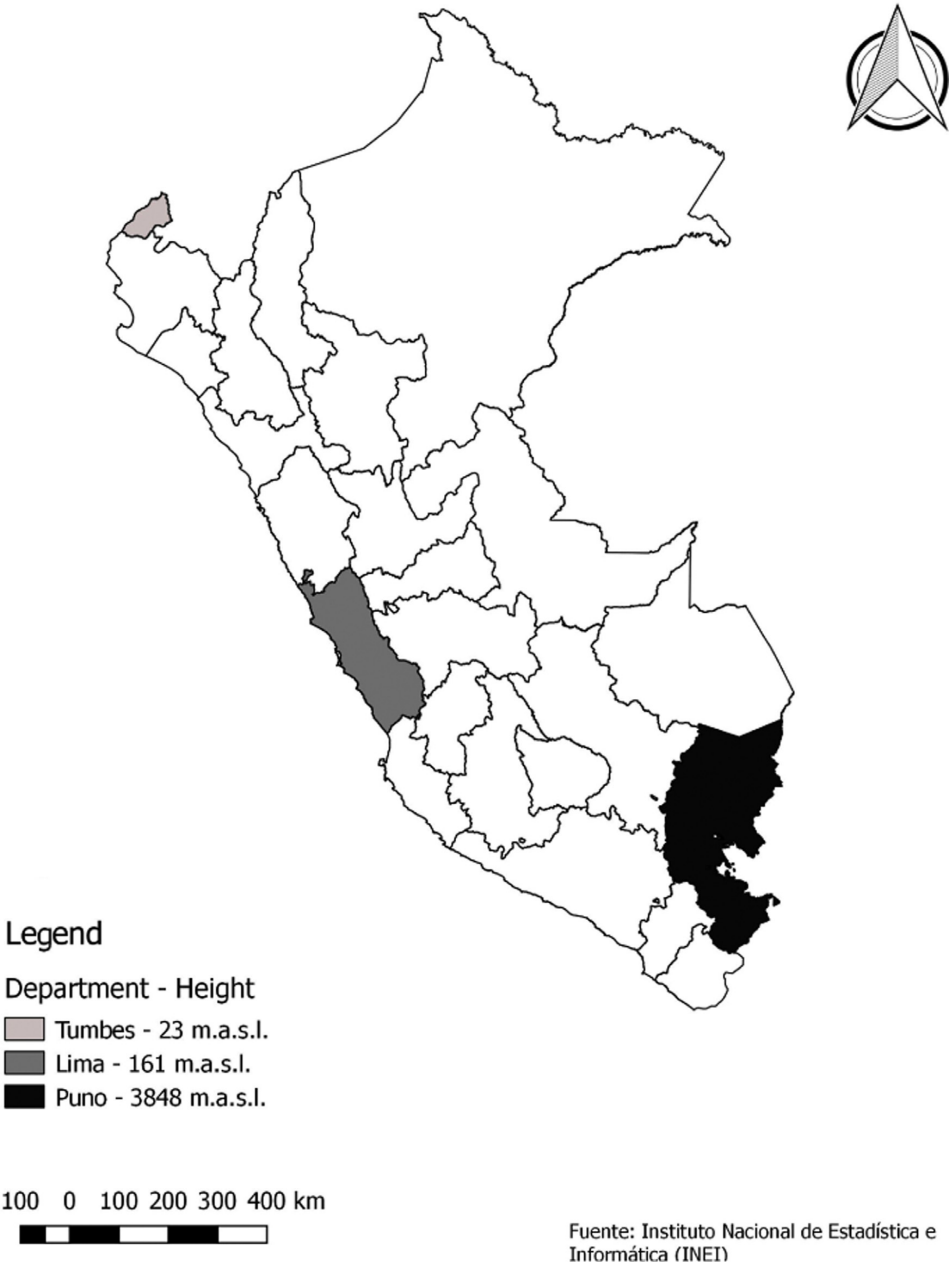
Map of Peru indicating the CRONICAS Cohort Study's sites.

Baseline information from the CRONICAS Cohort Study, collected in 2010-2011, was analyzed in the present study. The CRONICAS Cohort Study was conducted in 4 different settings: Pampas de San Juan de Miraflores, a highly urbanized community of approximately 15,000 inhabitants/km^2^ and located within Lima, the capital city of Peru, at sea level. Tumbes, a semi-urban site with 250 inhabitants/km^2^ is located in the northern coast of Peru, also at sea level. Puno, the high-altitude site, is located on the shore of Lake Titicaca at 3825 m.a.s.l. and contributed with an urban site and a rural site with population densities of 9940 inhabitants/km^2^ and 31 inhabitants/km^2^, respectively.^15^ (Fig. 1).

All participants were ≥35 years, full-time residents in the study area, and provided informed consent. Participants were excluded if they were pregnant, had any physical disability that would difficult take measurements, and had active pulmonary tuberculosis. A sex- and age-stratified (35-44, 45-54, 55-64, and ≥65 years) random sampling technique was conducted using the most updated census available in each site. In Puno, recruitment was stratified to include 500 participants in each of the urban and rural sites.^15^

### Variables definition

Six dyslipidemia traits were chosen as the main outcomes of interests. Three of them were selected because they are the most common dyslipidemia indicators that have been described to be associated with cardiovascular disease and mortality, including hypertriglyceridemia (TG ≥ 200 mg/dL), high low-density lipoprotein cholesterol (LDL-c ≥ 160 mg/dL), and low high-density lipoprotein cholesterol (HDL-c < 40 mg/dL in men and <50 mg/dL in women). A fourth indicator, high non-HDL-c (non-HDL-c ≥160 mg/dL), emerging as a new cardiovascular risk factor,^16^ was also evaluated in the whole sample. The 2 other dyslipidemia traits, nonisolated low HDL-c (low HDL-c accompanied by high TG and/or high LDL-c) and isolated low HDL-c (low HDL-c and TG < 200 mg/dL and LDL-c<160 mg/dL), correspond to a subgroup of low-HDL-c, a highly prevalent pattern of dyslipidemia as reported in previous studies in Peru and Latin America.^16^, ^17^

The exposures of interest were urbanization level (highly urban, urban, semi-urban, and rural) and altitude (high altitude vs sea level). It is important to clarify that highly urban and semi-urban areas were at sea level and urban and rural areas were at high altitude level. The urbanization level was defined for the population density 15,000; 9940; 250; and 31 inhabitants/km^2^.

Other variables included in the analysis were sociodemographic, lifestyle factors, body mass index (BMI), and comorbidities. Sociodemographic variables were sex, age, educational level (<7, 7–11, and ≥ 12 years), and socioeconomic status assessed through a wealth index derived from assets possession and household facilities, in tertiles. Lifestyle variables were current daily smoking was self-reported and defined as smoking at least one cigarette per day. Hazardous drinking was considered if the participant had a score ≥8 using the Alcohol Use Disorder Identification Test.^18^ Physical activity levels were measured combining leisure time and transportation-related physical activity domains of the International Physical Activity Questionnaire and classified as high/moderate vs low physical activity.^19^ Patterns of consumption of certain foods, each variable representing the daily consumption of several types of food, were divided into tertiles. BMI was included and categorized as normal (≥18.5 to <25 kg/m^2^), overweight (≥25 to <30 kg/m^2^), and obese (≥30 kg/m^2^). Clinical variables were hypertension, blood pressure was measured 3 times, systolic blood pressure (SBP) and diastolic blood pressure (DBP) were calculated from the average of the second and third measurements and hypertension was defined as having a SBP ≥140 mmHg, or DBP ≥90 mmHg, or self-report of physician diagnosis or use of antihypertensive medication. Diabetes was defined as having fasting blood glucose ≥126 mg/dL (≥7 mmol/L) or a self-report of physician diagnosis or use of antidiabetic medication, and impaired fasting glucose was defined as having fasting blood glucose between 110 and < 126 mg/dL.^20^

### Procedures

Fieldwork activities and procedures of the CRONICAS Cohort Study have been described in detail elsewhere.^15^ Trained community health workers applied face-to-face questionnaires. After completing the questionnaire, an appointment for a clinical assessment was arranged to ensure an adequate fasting period, between 8 and 12 hours, where a total of 13.5 mL of blood was drawn. Standing height was measured using standardized techniques. After a 5-minute resting period, blood pressure was measured using an automatic monitor OMRON HEM-780 (OMRON, Tokyo, Japan) validated for adult population.

For laboratory procedures, Cobas Modular Platform automated analyzer and reagents from Roche Diagnostics were used to measure triglycerides, total cholesterol, and HDL-c. Also, LDL-c was measured using this method in participants with triglycerides ≥400 mg/dL. However, if participants had triglycerides below 400 mg/dL, the Friedewald equation was used to calculate LDL-c in mg/dL. Non-HDL-c was also estimated by subtracting HDL-c from total cholesterol. Serum glucose was measured using an enzymatic colorimetric method (GOD-PAP; Modular P-E/Roche-Cobas, Grenzach-Wyhlen, Germany).

### Statistical analysis

All statistical analyses were performed using Stata 12.0 (Stata Corp, College Station, TX). Prevalence and 95% confidence intervals (95% CIs) of lipid profile patterns by altitude and level of urbanization were estimated. The chi-squared analysis was used to show the association between altitude, level of urbanization, sociodemographic, lifestyle factors, BMI, and comorbidities with dyslipidemia patterns.

Different models were generated to evaluate the crude and adjusted association of dyslipidemia patterns with our main exposures (urbanization level and altitude). We used Poisson regression with robust variance, and prevalence ratios and 95% CIs were reported.^21^ Our first models compared specifically: (1) hypertriglyceridemia vs normal triglycerides; (2) high LDL-c vs normal LDL-c; (3) low HDL-c vs normal HDL-c; (4) high non-HDL-c vs normal non-HDL-c and also the subgroups of low HDL-c; (5) isolated low HDL vs normal HDL-c; and (6) nonisolated low HDL-c vs normal HDL-c. Models were adjusted by different variables: model 1 was adjusted by sociodemographic variables such as age, sex, education, and wealth index; model 2 provided estimates adjusting for lifestyle factors and clinical variables, namely daily smoking, hazardous drinking, physical activity, hypertension, and diabetes. Model 3, in addition to the aforementioned variables, included adjustment for BMI as well as checking for collinearity. The analytical approach, including the selection of variables used for adjustment in the models, was decided using the criteria of prior information.^22^

### Ethics

The Institutional Review Boards of Universidad Peruana Cayetano Heredia and Asociación Benéfica PRISMA in Lima, Peru, and Johns Hopkins University in Baltimore, United States, approved the study. Participants received information about the objectives and procedures of the study and gave oral consent due to high rates of illiteracy, mainly in rural and semi-urban areas.

## Results

Response rate at baseline was 62.9% (4325/6872), and of these, 83.3% (3601/4325) had completed questionnaires. Among those with completed questionnaires, 84.3% (3037/3601) had complete lipid profile parameters for the analysis. The characteristics of the participants included vs those not included in the analysis are available in Supplementary Table 1. Differences in education level, socioeconomic status, hazardous drinking, physical activity, hypertension, and diabetes were found between the participants included compared with those not included in this analysis.

Data from 3037 participants, 48.5% males, mean age of 55.6 years (standard deviation [SD] ± 12.7) were used in the analyses. The characteristics of the study population according to study site are presented in the Supplementary Table 2. In these bivariate analyses, there was evidence of an association between study site and education level, socioeconomic status, daily smoking, hazardous drinking, physical activity, hypertension, and diabetes.

Mean level of triglycerides was 149.0 mg/dL (SD ± 70.0); LDL-c level was 127.2 mg/dL (SD ± 34.5); and HDL-c level was 41.9 mg/dL (SD ± 11.4), whereas the level of non-HDL-c was 156.9 mg/dL (39.5) with differences between study sites (Table 1).

**Table 1:** Means of the plasma lipids

	Total (N = 3037)	Sea level highly urban (N = 1005)	High-altitude urban (N = 506)	Sea level semi-urban (N = 991)	High-altitude rural (N = 535)	*P* value
Triglycerides, mean (SD)	149.0 (70.0)	154.8 (73.1)	159.3 (69.3)	149.4 (68.9)	127.7 (61.9)	<.001
LDL-c, mean (SD)	127.2 (34.5)	127.6 (33.4)	128.7 (36.7)	131.9 (33.8)	116.4 (33.1)	<.001
HDL-c, mean (SD)	41.9 (11.4)	41.3 (11.2)	41.7 (10.9)	41.2 (11.8)	44.4 (11.3)	<.001
Non-HDL-c, mean (SD)	156.9 (39.5)	158.5 (38.5)	160.6 (41.2)	161.7 (38.4)	141.9 (38.4)	<.001

HDL-c, high-density lipoprotein cholesterol; LDL-c, low-density lipoprotein cholesterol; SD, standard deviation.

Table 2, Table 3 show the bivariate association between socio demographic, lifestyle, BMI, and other clinical variables with dyslipidemia traits. Age and BMI were associated with all dyslipidemia patterns. Female sex was associated with high LDL-c, low HDL-c, and its subgroups. Also, hypertriglyceridemia, low HDL-c, and high LDL-c were associated with high socioeconomic status.

**Table 2:** Characteristics of the study population by dyslipidemia traits

	N	Hypertriglyceridemia		High LDL-c		Low HDL-c		High non-HDL-c	
		n (%)	*P* value	n (%)	*P* value	n (%)	*P* value	n (%)	*P* value
Sociodemographics									
Sex									
Female	1565	298 (19.0)	.08	291 (18.6)	<.001	1176 (75.1)	<.001	708 (45.2)	.33
Male	1472	318 (21.6)		206 (14.0)		802 (54.5)		640 (43.5)	
Age									
35– 44 y	742	131 (17.7)	<.001	88 (11.9)	<.001	527 (71.0)	<.001	289 (38.9)	<.001
45–54 y	771	181 (23.5)		135 (17.5)		509 (66.0)		359 (46.6)	
55–64 y	766	190 (24.8)		158 (20.6)		503 (65.7)		398 (51.9)	
65+ y	756	114 (15.1)		115 (15.2)		438 (57.9)		301 (39.8)	
Education level									
< 7 y	1394	237 (17.0)	<.001	209 (15.0)	.11	897 (64.4)	.28	567 (40.7)	.001
7–11 y	1001	201 (20.1)		169 (16.9)		646 (64.5)		469 (46.9)	
12+ y	640	178 (27.8)		119 (18.6)		434 (67.8)		312 (48.8)	
Socioeconomic status									
Lowest tertile	948	135 (14.2)	<.001	123 (13.0)	.003	591 (62.3)	.03	336 (35.4)	<.001
Middle tertile	1042	210 (20.2)		183 (17.6)		676 (64.9)		472 (45.3)	
Highest tertile	1047	271 (25.9)		191 (18.2)		711 (67.9)		540 (51.6)	
Lifestyle behaviors									
Daily smoking									
No	2939	593 (20.2)	.43	479 (16.3)	.59	1924 (65.5)	.03	1302 (44.3)	.61
Yes	98	23 (23.5)		18 (18.4)		54 (55.1)		46 (46.9)	
Hazardous drinking									
No	2625	517 (19.7)	.04	444 (16.9)	.04	1762 (67.1)	<.001	1170 (44.6)	.60
Yes	412	99 (24.0)		53 (12.9)		216 (52.4)		178 (43.2)	
Physical activity									
Low	972	196 (20.2)	.89	171 (17.6)	.18	651 (67.0)	.14	452 (46.5)	.10
Moderate/high	2061	420 (20.4)		323 (15.7)		1324 (64.2)		893 (43.3)	
Measurements									
Body mass index									
<25 kg/m^2^	904	81 (9.0)	<.001	105 (11.6)	<.001	408 (45.1)	<.001	255 (28.2)	<.001
≥25 and < 30 kg/m^2^	1322	306 (23.2)		229 (17.3)		913 (69.1)		643 (48.6)	
≥30 kg/m^2^	807	229 (28.4)		163 (20.2)		654 (81.0)		449 (55.6)	
Hypertension									
No	2253	445 (19.8)	.19	339 (15.1)	.001	1452 (64.5)	.19	953 (42.3)	<.001
Yes	780	171 (21.9)		158 (20.3)		523 (67.1)		394 (50.5)	
Diabetes									
No	2797	551 (19.7)	<.001	446 (16.0)	.03	1806 (64.6)	.03	1227 (43.9)	.05
Yes	240	65 (27.1)		51 (21.3)		172 (71.7)		121 (50.4)	

HDL-c, high-density lipoprotein cholesterol; LDL-c, low-density lipoprotein cholesterol; SD, standard deviation.

**Table 3:** Characteristics of the study population by subgroups of low HDL-c

	Isolated low HDL		Non-isolated low HDL	
	n (%)	*P*	n (%)	*P*
Sociodemographics				
Sex				
Female	760/1149 (66.1)	<.001	416/805 (51.7)	<.001
Male	481/1151 (41.8)		321/991 (32.4)	
Age				
35–44 y	365/580 (62.9)	<.001	162/377 (43.0)	<.001
45–54 y	290/552 (52.5)		219/481 (45.5)	
55–64 y	282/545 (51.7)		221/484 (45.7)	
65+ y	304/622 (48.9)		134/452 (29.7)	
Education level				
< 7 y	606/1103 (54.9)	.62	291/788 (36.9)	.001
7–11 y	397/752 (52.8)		249/604 (41.2)	
12+ y	237/443 (53.5)		197/403 (48.9)	
Socioeconomic status				
Lowest tertile	417/774 (53.9)	.79	174/531 (32.8)	<.001
Middle tertile	415/781 (53.1)		261/627 (41.6)	
Highest tertile	409/745 (54.9)		302/638 (47.3)	
Lifestyle behaviors				
Daily smoking				
No	1207/2222 (54.3)	.06	717/1732 (41.4)	.11
Yes	34/78 (43.6)		20/64 (31.3)	
Hazardous drinking				
No	1118/1981 (56.4)	<.001	644/1507 (42.7)	.001
Yes	123/319 (38.6)		93/289 (32.2)	
Physical activity				
Low	393/714 (55.0)	.49	258/579 (44.6)	.03
Moderate/high	848/1585 (53.4)		476/1213 (39.2)	
Measurements				
Body mass index				
<25 kg/m^2^	311/807 (38.5)	<.001	97/593 (16.4)	<.001
≥25 and < 30 kg/m^2^	550/959 (57.4)		363/772 (47.0)	
≥30 kg/m^2^	377/530 (71.1)		277/430 (64.4)	
Hypertension				
No	926/1727 (53.6)	.61	526/1327 (39.6)	.04
Yes	312/569 (54.8)		211/468 (45.1)	
Diabetes				
No	1138/2129 (53.5)	.09	668/1659 (40.3)	.02
Yes	103/171 (60.2)		69/137 (50.4)	

HDL, high-density lipoprotein; LDL, low-density lipoprotein.

### Lipid profiles by level of urbanization and altitude

**Figure 2:**
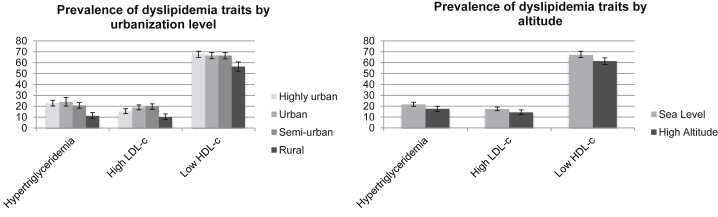
Prevalence of dyslipidemia traits by urbanization level and altitude. Hypertriglyceridemia (*P* < .001), high LDL-c (*P* < .001), and low HDL-c (*P* < .001); hypertriglyceridemia (*P* = .006), high LDL-c (*P* = .03), and low HDL-c (*P* = .002). HDL-c, high-density lipoprotein cholesterol; LDL-c, low-density lipoprotein cholesterol.

**Figure 3:**
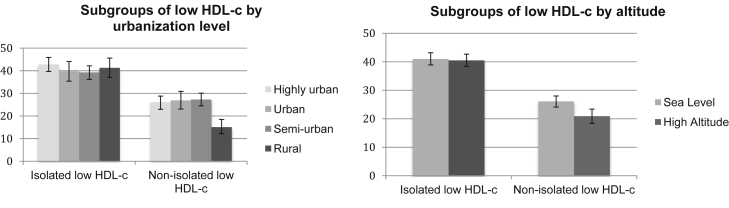
Prevalence of subgroups of low HDL-c by urbanization level and altitude. Isolated low HDL-c (*P* = .05) and nonisolated low HDL-c (*P* < .001); isolated low HDL-c (*P* = .05) and nonisolated low HDL-c (*P* < .001). HDL-c, high-density lipoprotein cholesterol; LDL-c, low-density lipoprotein cholesterol.

**Figure 4:**
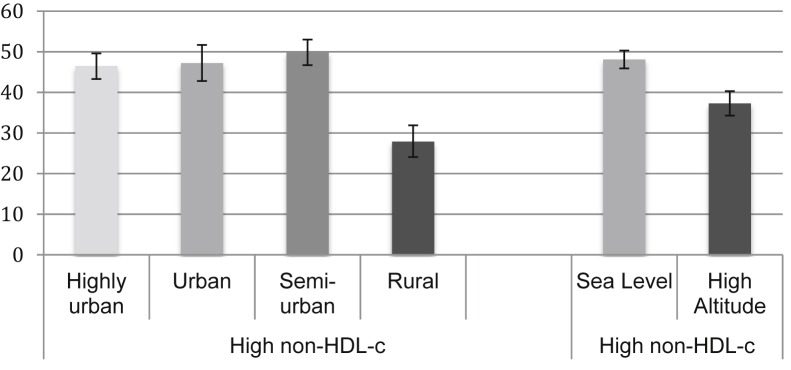
Prevalence of high non-HDL-c by urbanization level and altitude. Urbanization level (*P* < .001) and altitude (*P* < .001). HDL-c, high-density lipoprotein cholesterol.

High non-HDL-c was the most common lipid trait in all study sites followed by low HDL-c. In general, in terms of urbanization, a common pattern was observed where the rural site had the lowest prevalence of all lipid profiles studied compared with all other study sites, with the exception of isolated low HDL where no differences were observed. When both rural and urban Puno sites were combined into a high-altitude variable, the same pattern described before was observed, with the high altitude setting having a lower prevalence of lipid traits than the sea level sites. The prevalence of dyslipidemia traits by urbanization and altitude are shown in Figure 2, Figure 3, Figure 4, and the point prevalence estimates and 95% CIs are presented in Supplementary Table 3.

### Association between level of urbanization and lipid traits

In the crude analyses, we found evidence of an association between level of urbanization, especially in the rural site, and the 6 primary outcomes of interest. Further adjustment by sociodemographic, lifestyle factors, and clinical variables maintained the association in 5 out of the 6 dyslipidemia traits in the rural site.

Relative to the highly urban site, participants living in rural areas had 40% lower prevalence of hypertriglyceridemia and 6% lower prevalence of high non-HDL-c (Table 4, adjusted model 2). In addition, there was evidence of a 37% higher prevalence of high LDL-c among individuals living in the semi urban area relative to those in the highly urban area.

**Table 4:** Association between urbanization level/altitude and the prevalence of lipid patterns

	High-altitude rural (vs sea level highly urban)	High-altitude urban (vs sea level highly urban)	Sea level semi-urban (vs sea level highly urban)	High-altitude (vs sea level)
	PR (95% CI)			
Hypertriglyceridemia				
Crude	**0.49 (0.38–0.64)**	1.06 (0.87–1.28)	0.91 (0.77–1.07)	**0.80 (0.69–0.94)**
Adjusted model 1	**0.60 (0.45–0.81)**	0.96 (0.78–1.18)	0.98 (0.83–1.17)	**0.81 (0.69–0.95)**
Adjusted model 2	**0.60 (0.45–0.81)**	0.96 (0.78–1.18)	0.93 (0.78–1.12)	**0.83 (0.70–0.98)**
Adjusted model 3	**0.75 (0.56–0.99)**	0.95 (0.78–1.17)	0.92 (0.77–1.10)	0.91 (0.77–1.07)
High LDL-c (160)				
Crude	**0.66 (0.49–0.88)**	1.23 (0.97–1.55)	**1.28 (1.05–1.55)**	**0.82 (0.69–0.98)**
Adjusted model 1	0.78 (0.57–1.08)	1.15 (0.90–1.48)	**1.39 (1.14–1.69)**	**0.82 (0.68–0.99)**
Adjusted model 2	0.80 (0.58–1.11)	1.15 (0.90–1.48)	**1.37 (1.12–1.68)**	**0.85 (0.70–1.03)**
Adjusted model 3	0.86 (0.62–1.20)	1.16 (0.90–1.49)	**1.37 (1.11–1.67)**	0.88 (0.72–1.08)
Low HDL-c				
Crude	**0.83 (0.77–0.91)**	0.98 (0.91–1.06)	0.98 (0.92–1.05)	**0.92 (0.86–0.97)**
Adjusted model 1	**0.86 (0.78–0.94)**	0.97 (0.90–1.05)	0.99 (0.93–1.06)	**0.92 (0.87–0.97)**
Adjusted model 2	**0.87 (0.79–0.95)**	0.98 (0.91–1.07)	0.99 (0.93–1.06)	**0.93 (0.87–0.99)**
Adjusted model 3	0.97 (0.88–1.06)	0.98 (0.91–1.06)	0.99 (0.93–1.06)	0.98 (0.92–1.04)
High non-HDL-c				
Crude	**0.60 (0.51–0.69)**	1.02 (0.91–1.14)	1.07 (0.97–1.17)	**0.77 (0.71–0.85)**
Adjusted model 1	**0.69 (0.59–0.82)**	1.00 (0.89–1.13)	**1.15 (1.04–1.26)**	**0.79 (0.72–0.88)**
Adjusted model 2	**0.70 (0.59–0.83)**	1.00 (0.89–1.13)	**1.12 (1.01–1.24)**	**0.81 (0.74–0.90)**
Adjusted model 3	**0.79 (0.67–0.93)**	1.00 (0.89–1.13)	**1.11 (1.01–1.22)**	**0.86 (0.78–0.95)**
Subgroups of low HDL-c vs normal low HDL-c				
Isolated low HDL-c				
Crude	**0.85 (0.76–0.96)**	0.95 (0.85–1.07)	0.95 (0.86–1.04)	0.92 (0.85–1.00)
Adjusted model 1	**0.86 (0.76–0.97)**	0.96 (0.85–1.08)	0.95 (0.87–1.04)	0.93 (0.85–1.01)
Adjusted model 2	**0.87 (0.77–0.99)**	0.97 (0.86–1.09)	0.96 (0.87–1.06)	0.94 (0.86–1.02)
Adjusted model 3	0.97 (0.86–1.09)	0.97 (0.86–1.09)	0.95 (0.87–1.05)	0.99 (0.91–1.08)
Nonisolated low HDL-c				
Crude	**0.59 (0.48–0.73)**	1.03 (0.88–1.20)	1.03 (0.91–1.17)	**0.79 (0.70–0.90)**
Adjusted model 1	**0.69 (0.55–0.87)**	0.97 (0.82–1.15)	1.09 (0.96–1.25)	**0.80 (0.70–0.91)**
Adjusted model 2	**0.70 (0.56–0.88)**	0.97 (0.82–1.15)	1.07 (0.93–1.23)	**0.82 (0.72–0.94)**
Adjusted model 3	0.91 (0.73–1.12)	0.98 (0.84–1.15)	1.07 (0.93–1.22)	0.92 (0.81–1.04)

CI, confidence interval; HDL-c, high-density lipoprotein cholesterol; LDL-c, low-density lipoprotein cholesterol; PR, prevalence ratio.

Adjusted model 1: adjusted by sex, age, education, and wealth index.

Adjusted model 2: adjusted by sex, age, education, wealth index, hazardous drinking, physical activity, hypertension, and diabetes.

Adjusted model 3: adjusted by sex, age, education, wealth index, hazardous drinking, physical activity, hypertension, diabetes, and body mass index.

Bold stands out some prevalence ratio with a significant confidence interval.

### Association between altitude and lipid traits

We found evidence of an association between altitude and 5 out of the 6 primary outcomes focused on single dyslipidemia traits, with the exception of isolated low HDL. These associations remained present in the crude and non–BMI-adjusted models, even when sociodemographic, lifestyle factors, and clinical variables were included (Table 4).

### The role of BMI: BMI-adjusted models

In terms of urbanization, when BMI was included in the models, associations remained in the rural site for hypertriglyceridemia and high non-HDL-c outcomes, and for high LDL-c in the semi-urban area. In the case of altitude, additional adjustment for BMI attenuated all the estimates and the association disappeared in all cases with the exception of high non-HDL-c (Table 3, adjusted model 3). The addition of BMI to these models did not show evidence of collinearity with the other variables used for adjustment.

## Discussion

### Main findings

Our findings show that it was largely urbanization, particularly rurality rather than altitude, the main driver in the association with dyslipidemia traits. The analysis pooling data into high-altitude vs sea level sites do carry the predominant associations observed in the rural high-altitude site. This observation favors the interpretation that it is urbanization the exposure that is strongest associated with the outcomes of interest.

### Comparison with other studies

The study of dyslipidemia according to urbanization and altitude as joint exposures and within the same population is limited. Most studies have compared rural vs urban areas without considering the effect of altitude. For example, studies in India and Peru reporting higher rates of isolated low HDL-c in rural in comparison to urban populations,^17^, ^23^ not confirmed in our study, and associations between hypercholesterolemia, hypertriglyceridemia, and high LDL-c with urban residence.^23^ Among the later, we were able to replicate the association with hypercholesterolemia and hypertriglyceridemia but not high LDL-c. One potential explanation lies on the fact that not all rural areas are necessarily identical, and although they share some commonalities in terms of population size, it is possible that lifestyle across rural and urban areas carries other differences, particularly in relation to diet,^10^ physical activity,^11^ or other factors,^24^ which could alter the relationship with the lipid-related outcomes of interest. In relation to dietary patterns, a Peruvian national nutritional survey has reported that individuals from Lima, our highly urban site, had a slightly lower consumption of fried foods in comparison to the rest of the coast but higher consumption than people from rural highlands,^25^ whereas with respect to protein intake, Lima had higher consumption in comparison to the rest of the country.^26^ It is also known that blood lipids are highly susceptible to the intake of protein and carbohydrates, with higher levels of triglycerides and LDL-c among those with lower protein intake, despite the similar amount of carbohydrates consumed.^27^, ^28^

With regard to altitude and lipid profiles, previous studies have compared high-altitude vs sea level population. Previous studies from Peru have included rural populations living at 4100 m.a.s.l. vs urban sea level groups,^5^ as well as groups at ≥3000 m.a.s.l. vs < 1000 m.a.s.l.^7^ These studies showed crude estimates and did not adjust by potential confounders nor by BMI, limiting the comparability with our findings. A separate study from Oman, in the Arabian peninsula, compared HDL-c levels in families living at different altitudes, that is, 2000 vs 700 m.a.s.l., and found that this marker was lower in the population living at higher altitude in the order of –0.39 mmol/L (15 mg/dL),^8^ a difference that may not have much clinical relevance. In our study, we did not observe an association between low HDL-c and altitude.

We built different models to explore our association of interest, including a final model that adjusted by BMI. In doing so, such adjustment markedly reduced the strength of the associations, attenuating toward the null, the majority of them.

BMI is independently associated with patterns of dyslipidemia and also with level of urbanization and altitude; for these reasons, BMI was treated as a confounder. It is well known that obesity causes high LDL-c, hypertriglyceridemia, and low HDL-c.^29^ In terms of the relationship between BMI and our exposures of interest, previous studies have found that obesity increases with some aspects of urbanization like lower diet quality scores and less physical activity,^30^ and longitudinal studies have found that urban populations have a 9-time increased risk of developing obesity relative to rural population.^31^ With regard to altitude, a study from Spain reported that living at higher altitude was inversely associated with the risk of developing overweight or obesity.^32^ Although some may consider that obesity lies in the causal pathway of the association between our exposures of interest and lipid profiles, we decided to maintain the adjustment for BMI as separate results, so that it can guide the assessment of our estimates and ensure comparability with other studies.

### Potential explanation to our findings

The basis to explore altitude as a predictor of alterations in lipid profiles has a physiological basis. Periodic hypoxemia produces an alteration in the oxidation of lipids in the hepatic cells,^33^ and acute exposure to high altitude found increased levels of HDL and decreased levels of triglycerides.^34^ One of the challenges observed is that unraveling the effects of altitude on lipid profiles is difficult given the predominance of studies focused on rural high-altitude areas only, without the assessment of urban high-altitude settings. Our study capitalizes on the opportunity of studying both urban and rural sites at the same level of altitude, thus removing the effect of urbanization on our association of interest. Hence, we contend that it is rurality, but not altitude, the main driver of the associations observed. It is possible that lifestyle factors associated with urbanization may largely explain our findings rather than physiological parameters observed at high altitude. For instance, most of the dyslipidemia traits studied had a lower prevalence in the rural population than in the urban one.

### Strengths and limitations

The CRONICAS Cohort Study affords us the study of a combination of different sites by their specific features of altitude and urbanization, thus providing a unique opportunity to test our hypothesis. However, we only compare 2 different levels of altitude where high altitude includes urban and rural populations, and sea level included semi-urban and highly urbanized populations. Nevertheless, our study is one of the few studies that evaluates the association of patterns of lipid profile among people living different levels of urbanization and altitude. Other limitations are as follows: first, it may be argued that the genetic background may have a role in determining the differences observed. However, our results are not likely to be fully explained by different genetic backgrounds. The genetic admixture in Peruvians is very high^35^ with many groups sharing common Native American ancestry, and the European ancestry component is relatively small (<10%).^36^ This decreases, but does not eliminate, the role that genetics may play in the associations observed. Even if genetics had a role, this is likely to be superseded by the effect of lifestyle factors on lipid profiles.^37^ Second, it has been described that the environment is related to lifestyle and dietary patterns,^38^, ^39^ and we did not explore dietary patterns in our study populations. A more detailed diet assessment would have informed our study; yet, conducting such measurements in large population-based studies is not always feasible nor free from limitations.^40^ Third, some selection bias may be present in our analysis as participants with data available for the analysis differed in certain socioeconomic characteristics from those who did not contribute data to the analysis. Finally, population density is a criterion to define different urbanization levels. However, in many countries some additional criteria are necessary to define different degrees of urbanization.^41^

## Conclusions

Levels of urbanization but not altitude were associated with dyslipidemia traits. People living in rural areas had a lower prevalence of dyslipidemia traits than their urban counterparts. In the case of the population from semi-urban areas, they showed a higher prevalence of high LDL-c and non-HDL-c than the highly urban area. Taken together, these finding supports discarding a relationship between high altitude and lipid traits but rather placing emphasis on urbanization as a key factor linked to lipid patterns.

## Figures and Tables

**Supplementary Table 1:** Characteristics of study population included and not included in the analysis

	Included (N = 3037) (%)	Not included (N = 582) (%)	*P* value
Sociodemographics			
Sex			
Female	1565 (84.5)	287 (15.5)	.90
Male	1472 (84.4)	273 (15.6)	
Age			
35–44 y	742 (86.5)	116 (13.5)	.28
45–54 y	771 (83.6)	151 (16.4)	
55–64 y	766 (83.5)	151 (16.5)	
65+ y	756 (84.0)	144 (16.0)	
Education level			
< 7 y	1394 (84.8)	249 (15.2)	<.001
7–11 y	1001 (86.7)	154 (13.3)	
12+ y	640 (80.0)	160 (20.0)	
Socioeconomic status			
Lowest tertile	948 (78.9)	253 (21.1)	<.001
Middle tertile	1042 (86.8)	159 (13.2)	
Highest tertile	1047 (87.3)	152 (12.7)	
Lifestyle behaviors			
Daily smoking			
No	2939 (84.2)	551 (15.8)	.107
Yes	98 (89.9)	11 (10.1)	
Hazardous drinking			
No	2625 (85.2)	457 (14.8)	.001
Yes	412 (79.5)	106 (20.5)	
Physical activity			
Low	972 (87.3)	142 (12.8)	.001
Moderate/high	2061 (83.1)	420 (16.9)	
Measurements			
Body mass index			
≥18.5 to <25 kg/m^2^	904 (94.8)	50 (5.2)	.24
≥25 and < 30 kg/m^2^	1322 (93.8)	88 (6.2)	
≥30 kg/m^2^	807 (92.9)	62 (7.1)	
Hypertension			
No	2253 (94.0)	143 (5.9)	<.001
Yes	780 (88.8)	98 (11.2)	
Diabetes			
No	2797 (97.3)	78 (2.7)	<.001
Yes	240 (87.6)	34 (12.4)	

**Supplementary Table 2:** Characteristics of study population according to urbanization level

	Sea level highly urban (N = 1005) (%)	High-altitude urban (N = 506) (%)	Sea level semi-urban (N = 991) (%)	High-altitude rural (N = 535) (%)	*P* value
Sociodemographics					
Sex					
Female	552 (51.9)	262 (51.8)	502 (50.7)	279 (52.2)	.93
Male	483 (48.1)	244 (48.2)	489 (49.3)	256 (47.8)	
Age					
35–44 y	242 (24.1)	127 (25.1)	251 (25.3)	122 (22.9)	.96
45–54 y	270 (26.9)	125 (24.7)	241 (24.3)	135 (25.3)	
55–64 y	252 (25.1)	129 (25.5)	249 (25.1)	136 (25.5)	
65+ y	241 (23.9)	125 (24.7)	250 (25.2)	140 (26.3)	
Education level					
< 7 y	431 (42.9)	75 (14.8)	551 (55.7)	337 (63.0)	<.001
7–11 y	399 (39.7)	139 (27.5)	300 (30.3)	163 (30.5)	
12+ y	174 (17.3)	292 (57.7)	139 (14.0)	35 (6.5)	
Socioeconomic status					
Lowest tertile	122 (12.1)	118 (23.3)	324 (32.7)	384 (71.8)	<.001
Middle tertile	366 (36.4)	132 (26.1)	406 (41.0)	138 (25.8)	
Highest tertile	517 (51.5)	256 (50.6)	261 (26.3)	13 (2.4)	
Language mainly spoken at home					
Spanish	978 (97.3)	485 (96.0)	990 (99.9)	67 (12.5)	<.001
Quechua	26 (2.6)	16 (3.2)	1 (0.1)	79 (14.7)	
Aymara	1 (0.1)	4 (0.8)	0 (0)	389 (72.7)	
Lifestyle behaviors					
Daily smoking					
No	972 (96.7)	495 (97.8)	938 (94.7)	534 (99.8)	<.001
Yes	33 (3.3)	11 (2.2)	53 (5.4)	1 (0.2)	
Hazardous drinking					
No	884 (88.0)	415 (82.0)	883 (89.1)	443 (82.8)	<.001
Yes	121 (12.0)	91 (18.0)	108 (10.9)	92 (17.2)	
Physical activity					
Low	190 (18.9)	108 (21.4)	537 (54.2)	137 (25.7)	<.001
Moderate/high	814 (81.1)	396 (78.6)	454 (45.8)	397 (74.3)	
Measurements					
Body mass index					
≥18.5 to <25 kg/m2	238 (23.7)	122 (24.3)	249 (25.1)	295 (55.2)	<.001
≥25 and < 30 kg/m2	452 (45.0)	251 (49.9)	431 (43.5)	188 (35.2)	
≥30 kg/m^2^	315 (31.3)	130 (25.8)	311 (31.4)	51 (9.6)	
Hypertension					
No	729 (72.5)	397 (78.9)	677 (68.3)	450 (84.3)	<.001
Yes	276 (27.5)	106 (21.1)	314 (31.7%)	84 (15.7)	
Dysglycemia					
No	871 (86.7)	442 (87.4)	829 (83.7)	503 (94.0)	<.001
Impaired fasting glucose	58 (5.8)	25 (4.9)	58 (5.9)	11 (2.1)	
Diabetes	76 (7.6)	39 (7.7)	104 (10.5)	21 (3.9)	
Diet					
Legumes					
Lowest	273 (27.2)	357 (70.5)	110 (11.1)	366 (68.4)	<.001
Middle	482 (47.9)	121 (23.9)	576 (58.1)	111 (20.8)	
Highest	250 (24.9)	28 (5.5)	305 (30.8)	58 (10.8)	
Fried food					
Lowest	615 (61.2)	283 (55.9)	540 (54.5)	315 (58.9)	<.001
Middle	267 (26.6)	127 (25.1)	231 (23.3)	124 (23.2)	
Highest	123 (12.2)	96 (18.9)	220 (22.2)	96 (17.9)	
Fruits					
Lowest	372 (37.0)	199 (39.3)	497 (50.2)	416 (77.8)	<.001
Middle	428 (42.6)	237 (46.8)	411 (41.5)	113 (21.1)	
Highest	205 (20.4)	70 (13.8)	83 (8.4)	6 (1.1)	
Processed sugar					
Lowest	464 (46.2)	312 (61.7)	77 (7.8)	509 (95.1)	<.001
Middle	519 (51.6)	187 (36.9)	911 (91.95)	23 (4.35)	
Highest	22 (2.2)	7 (1.4)	3 (0.6)	3 (0.35)	
Meat					
Lowest	513 (51.0)	184 (36.4)	385 (38.9)	214 (40.0)	<.001
Middle	348 (34.6)	144 (28.5)	524 (52.9)	146 (27.3)	
Highest	144 (14.3)	178 (35.2)	82 (8.3)	175 (32.7)	

**Supplementary Table 3:** Prevalence of lipid profile patterns by study site

	N	Hypertriglyceridemia, n (%)*		High LDL-c (160), n (%)*		High LDL-c (130), n (%)*		Low HDL-c, n (%)		High non-HDL-c, n (%)	
		95% CI	*P* value	95% CI	*P* value	95% CI	*P* value	95% CI	*P* value	95% CI	*P* value
Altitude											
Sea level	1996	434 (21.7)	.006	348 (17.4)	.03	947 (47.4)	<.001	1339 (67.1)	.002	1996 (48.1)	<.001
		19.9%–23.6%		15.8%–19.2%		45.2%–49.7%		64.7%–70.5%		45.9%–50.3%	
High altitude	1041	182 (17.5)		149 (14.3)		389 (37.4)		639 (61.4)		1041 (37.3)	
		15.2%–19.9%		12.2%–16.6%		34.4%–40.4%		58.3%–64.4%		34.3%–40.3%	
Urbanization level											
Highly Urban	1005	229 (22.8)	<.001	154 (15.3)	<.001	451 (44.9)	<.001	680 (67.7)	<.001	1005 (46.5)	<.001
		20.2%–25.5%		13.2%–17.7%		41.8%–48.0%		64.7%–70.5%		43.3%–49.6%	
Urban	506	122 (24.1)		95 (18.8)		226 (44.7)		337 (66.6)		506 (47.2)	
		20.4%–28.1%		15.5%–22.4%		40.3%–49.1%		62.3%–70.7%		42.8%–51.7%	
Semi-urban	991	205 (20.7)		194 (19.6)		496 (50.1)		659 (66.5)		991 (49.7)	
		18.2%–23.3%		17.1%–22.2%		46.9%–53.2%		63.5%–69.4%		46.6%–52.9%	
Rural	535	60 (11.2)		54 (10.1)		163 (30.5)		302 (56.5)		535 (27.9)	
		8.7%–14.2%		7.7%–12.9%		26.6%–34.6%		52.1%–60.7%		24.1%–31.9%	

	N	Isolated low HDL, n (%)*	*P* value	Nonisolated low HDL, n (%)*	*P* value
		95% CI		95% CI	
Altitude					
Sea level	1996	819 (41.0)	.05	520 (26.1)	<.001
		38.9%–43.2%		24.1%–28.0%	
High altitude	1041	422 (40.5)		217 (20.9)	
		37.5%–43.6%		18.4%–23.4%	
Urbanization level					
Highly urban	1005	430 (42.8)	.05	250 (24.9)	<.001
		39.7%–45.9%		22.2%–27.7%	
Urban	506	201 (39.7)		136 (26.9)	
		35.4%–44.1%		23.1%–30.9%	
Semi-urban	991	389 (39.3)		270 (27.3)	
		36.2%–42.4%		24.5%–30.1%	
Rural	535	221 (41.3)		81 (15.1)	
		37.1%–45.6%		12.2%–18.5%	

CI, confidence interval; HDL-c, high-density lipoprotein cholesterol; LDL-c, low-density lipoprotein cholesterol.

*Column with participants without each dyslipidemia patterns is not shown.

## References

[1] Global Burden of Disease Study C (2015). Global, regional, and national incidence, prevalence, and years lived with disability for 301 acute and chronic diseases and injuries in 188 countries, 1990-2013: a systematic analysis for the Global Burden of Disease Study 2013. Lancet.

[2] Vinueza R., Boissonnet C.P., Acevedo M. (2010). Dyslipidemia in seven Latin American cities: CARMELA study. Prev Med.

[3] Howard B.V., Ruotolo G., Robbins D.C. (2003). Obesity and dyslipidemia. Endocrinol Metab Clin North Am.

[4] Mohanna S., Baracco R., Seclen S. (2006). Lipid profile, waist circumference, and body mass index in a high altitude population. High Alt Med Biol.

[5] Baracco R., Mohanna S., Seclen S. (2007). A comparison of the prevalence of metabolic syndrome and its components in high and low altitude populations in peru. Metab Syndr Relat Disord.

[6] Sherpa L.Y., Deji, Stigum H., Chongsuvivatwong V. (2011). Lipid profile and its association with risk factors for coronary heart disease in the highlanders of Lhasa, Tibet. High Alt Med Biol.

[7] Pajuelo-Ramirez J., Sánchez-Abanto J., Arbañil-Huamán H. (2010). Las enfermedades crónicas no transmisibles en el Perú y su relación con la altitud. Rev Soc Per Med Interna.

[8] Al Riyami N.B., Banerjee Y., Al-Waili K. (2015). The Effect of Residing Altitude on Levels of High-Density Lipoprotein Cholesterol: A Pilot Study From the Omani Arab Population. Angiology.

[9] Ochoa-Aviles A., Verstraeten R., Lachat C. (2014). Dietary intake practices associated with cardiovascular risk in urban and rural Ecuadorian adolescents: a cross-sectional study. BMC Public Health.

[10] Chee S.S., Ismail M.N., Ng K.K., Zawiah H. (1997). Food intake assessment of adults in rural and urban areas from four selected regions in Malaysia. Malays J Nutr.

[11] Masterson Creber R.M., Smeeth L., Gilman R.H., Miranda J.J. (2010). Physical activity and cardiovascular risk factors among rural and urban groups and rural-to-urban migrants in Peru: a cross-sectional study. Rev Panam Salud Publica.

[12] (2014). Rural Population in Latin America and Caribbean [Internet].

[13] Leon-Velarde F., Maggiorini M., Reeves J.T. (2005). Consensus statement on chronic and subacute high altitude diseases. High Alt Med Biol.

[14] West J. (2001). Altitude Encyclopedia.com: The Oxford Companion to the Body. http://www.encyclopedia.com/earth-and-environment/geology-and-oceanography/geology-and-oceanography/altitude.

[15] Miranda J.J., Bernabe-Ortiz A., Smeeth L., Gilman R.H., Checkley W., Group C.C.S. (2012). Addressing geographical variation in the progression of non-communicable diseases in Peru: the CRONICAS cohort study protocol. BMJ Open.

[16] National Cholesterol Education Program Expert Panel on Detection E, Treatment of High Blood Cholesterol in A (2002). Third Report of the National Cholesterol Education Program (NCEP) Expert Panel on Detection, Evaluation, and Treatment of High Blood Cholesterol in Adults (Adult Treatment Panel III) final report. Circulation.

[17] Lazo-Porras M., Bernabe-Ortiz A., Malaga G. (2016). Low HDL cholesterol as a cardiovascular risk factor in rural, urban, and rural-urban migrants: PERU MIGRANT cohort study. Atherosclerosis.

[18] (2001). AUDIT Cuestionario de Identificación de los Transtornos debidos al Consumo de Alcohol.

[19] (2005). Guidelines for Data Processing and Analysis of the International Physical Activity Questionnaire (IPAQ) short and Long Forms.

[20] American Diabetes A (2014). Diagnosis and classification of diabetes mellitus. Diabetes care.

[21] Barros A.J., Hirakata V.N. (2003). Alternatives for logistic regression in cross-sectional studies: an empirical comparison of models that directly estimate the prevalence ratio. BMC Med Res Methodol.

[22] Greenland S. (1989). Modeling and Variable Selection in Epidemiologic Analysis. Am J Public Health.

[23] Joshi S.R., Anjana R.M., Deepa M. (2014). Prevalence of dyslipidemia in urban and rural India: the ICMR-INDIAB study. PLoS One.

[24] Padrao P., Silva-Matos C., Damasceno A., Lunet N. (2011). Association between tobacco consumption and alcohol, vegetable and fruit intake across urban and rural areas in Mozambique. J Epidemiol Community Health.

[25] (2006). Encuesta Nacional de Indicadores Nutricionales, Bioquímicos, Socioeconómicos y Culturales Relacionados con las Enfermedades Crónicas Degenerativas.

[26] (2012). Peru: Consumo Per Cápita de los Principales alimentos 2008-2009.

[27] Parker B., Noakes M., Luscombe N., Clifton P. (2002). Effect of a high-protein, high-monounsaturated fat weight loss diet on glycemic control and lipid levels in type 2 diabetes. Diabetes Care.

[28] Layman D.K., Boileau R.A., Erickson D.J. (2003). A reduced ratio of dietary carbohydrate to protein improves body composition and blood lipid profiles during weight loss in adult women. J Nutr.

[29] Klop B., Elte J.W., Cabezas M.C. (2013). Dyslipidemia in obesity: mechanisms and potential targets. Nutrients.

[30] Delisle H., Ntandou-Bouzitou G., Agueh V., Sodjinou R., Fayomi B. (2012). Urbanisation, nutrition transition and cardiometabolic risk: the Benin study. Br J Nutr.

[31] Carrillo-Larco R.M., Bernabe-Ortiz A., Pillay T.D. (2016). Obesity risk in rural, urban and rural-to-urban migrants: prospective results of the PERU MIGRANT study. Int J Obes.

[32] Diaz-Gutierrez J., Martinez-Gonzalez M.A., Pons Izquierdo J.J., Gonzalez-Muniesa P., Martinez J.A., Bes-Rastrollo M. (2016). Living at higher altitude and incidence of overweight/obesity: prospective analysis of the SUN Cohort. PLoS One.

[33] Meerson F.Z., Tverdokhlib V.P., Nikonorov A.A., Filippov V.K., Frolov B.A. (1988). The role of suppression of cholesterol 7-hydroxylase activity of the liver in the development of atherogenic stress-induced dyslipoproteinemia. Kardiologiia.

[34] Ferezou J., Richalet J.P., Serougne C., Coste T., Wirquin E., Mathe D. (1993). Reduction of postprandial lipemia after acute exposure to high altitude hypoxia. Int J Sports Med.

[35] Ruiz-Linares A., Adhikari K., Acuna-Alonzo V. (2014). Admixture in Latin America: geographic structure, phenotypic diversity and self-perception of ancestry based on 7,342 individuals. PLoS Genet.

[36] Mao X., Bigham A.W., Mei R. (2007). A genomewide admixture mapping panel for Hispanic/Latino populations. Am J Hum Genet.

[37] Pollin T.I., Quartuccio M. (2013). What We Know About Diet, Genes, and Dyslipidemia: Is There Potential for Translation?. Curr Nutr Rep.

[38] Tripathy J.P., Thakur J.S., Jeet G., Chawla S., Jain S., Prasad R. (2016). Urban rural differences in diet, physical activity and obesity in India: are we witnessing the great Indian equalisation? Results from a cross-sectional STEPS survey. BMC Public Health.

[39] Eckert S., Kohler S. (2014). Urbanization and health in developing countries: a systematic review. World Health Popul.

[40] Shim J.S., Oh K., Kim H.C. (2014). Dietary assessment methods in epidemiologic studies. Epidemiol Health.

[41] (2017). Population density and urbanization United Nations.

